# Activation of GABA_B_ receptors inhibits protein kinase B /Glycogen Synthase Kinase 3 signaling

**DOI:** 10.1186/1756-6606-5-41

**Published:** 2012-11-28

**Authors:** Frances Fangjia Lu, Ping Su, Fang Liu, Zafiris J Daskalakis

**Affiliations:** 1 Centre for Addiction and Mental Health, 250 College Street, Toronto, ON, M5T 1, Canada; 2 Departments of Psychiatry, University of Toronto, Toronto, ON, M5T 1R8, Canada

## Abstract

Accumulated evidence has suggested that potentiation of cortical GABAergic inhibitory neurotransmission may be a key mechanism in the treatment of schizophrenia. However, the downstream molecular mechanisms related to GABA potentiation remain unexplored. Recent studies have suggested that dopamine D2 receptor antagonists, which are used in the clinical treatment of schizophrenia, modulate protein kinase B (Akt)/glycogen synthase kinase (GSK)-3 signaling. Here we report that activation of GABA_B_ receptors significantly inhibits Akt/GSK-3 signaling in a β-arrestin-dependent pathway. Agonist stimulation of GABA_B_ receptors enhances the phosphorylation of Akt (Thr-308) and enhances the phosphorylation of GSK-3α (Ser-21)/β (Ser-9) in both HEK-293T cells expressing GABA_B_ receptors and rat hippocampal slices. Furthermore, knocking down the expression of β-arrestin2 using siRNA abolishes the GABA_B_ receptor-mediated modulation of GSK-3 signaling. Our data may help to identify potentially novel targets through which GABA_B_ receptor agents may exert therapeutic effects in the treatment of schizophrenia.

## Introduction

Schizophrenia (SCZ) is a debilitating disorder that exacts enormous personal, social and economic costs. Accumulated evidence has suggested that potentiation of cortical GABAergic inhibitory neurotransmission may be a novel treatment target for resistant SCZ. The human GABA_B_ receptor gene has been localized to regions in the genome associated with schizophrenia, 6p21.3 [1,2]. In addition, the expression of the GABA_B_ receptor has been shown to be reduced in the human schizophrenic brain [3]. As well, the GABA_B_ receptor agonist, baclofen has been reported to have some efficacy in SCZ patients [4]. Baclofen was also shown to improve cognition in an animal model of methamphetamine-induced psychosis [5] and elicit antipsychotic-like effects in the rat paradigm of prepulse inhibition of the startle response, an animal phenotype for modeling SCZ [6].

Transcranial magnetic stimulation (TMS) indices of GABA_B_ receptor mediated inhibitory neurotransmission can be altered through antipsychotic treatment. The cortical silent period (CSP) represents a TMS neurophysiological index of GABA_B_ receptor mediated inhibitory neurotransmission whereas short interval cortical inhibition (SICI) represents a TMS neurophysiological index of GABA_A_ receptor mediated inhibitory neurotransmisssion. Both the CSP and SICI were lowered in patients with SCZ [7,8]. Clozapine treated patients demonstrated significantly longer CSP durations of large effect (i.e., Cohen’s D > 3) but no change in SICI relative to unmedicated SCZ patients and healthy subjects [9]. These findings suggest that clozapine potentiates the GABA_B_ receptor and also underscores the possibility that the GABA_B_ receptor may play a key role in the treatment of SCZ. Furthermore, a recent in-vivo study by Wu et al. also confirmed these findings [10] which reported that the binding of the GABA_B_ receptor antagonist ^3^H]-CGP54626A increased when treated with clozapine. There was a significant correlation between the clozapine dose and the increase of ^3^H]-CGP54626A binding in linear regression analysis. In the presence of clozapine, a left shift was shown for specific ^3^H]-CGP54626A binding in competition with different concentrations of GABA. Clozapine also increased ^3^H]-CGP54626A binding at GABA_B_ R1 subunit when HEK293 cells overexpressed GABA_B_ receptors, highlighting a potential therapeutic target for clozapine.

GSK-3 is a protein kinase originally identified and named for its ability to phosphorylate and inactivate the metabolic enzyme glycogen synthase [11]. Subsequently, GSK-3 was found to be broadly involved in neural systems and modulate many aspects of neuronal function, including gene expression, neurogenesis, synaptic plasticity, neuronal structure, and neuronal death and survival [12-14]. Accumulating evidence implicates abnormal activity of GSK-3 in psychiatric disorders, such as bipolar disorder, depression, schizophrenia, ADHD and Alzheimer’s Disease [15-17] and GSK-3 is a potential protein kinase target for antipsychotics. Atypical antipsychotics, such as clozapine and olanzapine, can regulate phospho-serine-GSK-3 and inhibit its activity [18].

There are two highly homologous GSK-3 enzymes, GSK-3α and GSK-3β, derived from separate genes. Both GSK-3α and GSK-3β are expressed throughout the brain [19] and they are regulated by several mechanisms. The most well-defined regulatory mechanism is by phosphorylation of serine-9 in GSK-3β or serine-21 in GSK-3α, which inhibits GSK-3 activity [20-22]. The Akt signaling pathway often is a major regulator of GSK-3 because Akt phosphorylates GSK-3 on these inhibitory serine residues, which has been shown to involved in dopamine signaling and many aspects of psychiatric disorders [23]. Conversely, enzymatic activity is enhanced by phosphorylation of tyrosine-216 in GSK-3β and tyrosine-279 in GSK-3α, which are autophosphorylation sites, and can facilitate substrate binding to GSK-3, although the mechanism of this modification are not well-defined [24].

The fact that all current antipsychotic drugs exert their effect through the blockade of dopamine D2 receptors (D2R) has established that increased D2R signaling is an important part of the pathophysiology of schizophrenia [25,26]. Recent studies have suggested that D2R can activate the Akt/GSK-3 pathway via G protein-independent signaling [20,27]. D2R-mediated Akt/GSK-3 regulation involves the recruitment of β-arrestin2 to the D2R and specific dephosphorylation/inactivation of the serine/threonine kinase Akt on its regulatory Thr-308 residue but not the second regulatory residue (Ser-473) [20]. Phosphorylation of Akt in response to DA leads to a reduction of kinase activity and a concomitant activation of its substrates GSK-3α (Ser-21)/β (Ser-9) [20]. More importantly, antipsychotics including haloperidol, clozapine and olanzapine strongly decrease recruitment of β-arrestin2 to D2R [18,28,29]. These data support a critical role of D2R-mediated GSK-3 signaling in the pathology of schizophrenia and suggest that antipsychotics exert their therapeutic effect by targeting GSK-3 signaling. Therefore, we investigated whether activation of GABA_B_ receptors can modulate GSK-3 signaling. This will be a step towards establishing the relationship between the GABA_B_ receptor and downstream targets of antipsychotic action, and potentially identifying new therapeutic targets for schizophrenia.

## Materials and methods

### Material

The cDNAs encoding human GABA_B_R1a and GABA_B_R2 subunits in pcDNA3 were kindly supplied by Dr. O. Carter Snead in The Hospital for Sick Children in Toronto.

The β-arrestin2 siRNA targeting human β-arrestin2 were purchased from Santa Cruz Biotechnology (cat# sc-29208).

### Cell culture and transient transfection

HEK293T cells were cultured in α-MEM ( Invitrogen, Carlsbad, CA) supplemented with 10% fetal bovine serum (Invitrogen) and maintained in incubators at 37°C, 5% CO_2_. HEK293T cells were grown to 90% confluence before being transiently transfected with plasmid constructs and/or siRNA using X-treme GENE 9 DNA transfection reagents (Roche). About 24–48 hours after transfection, cells were used for experiments.

### Protein extracts isolation

Transfected HEK293T cells were collected, washed with 1 × PBS, and solubilized with the buffer (50 mM Tris–HCl, pH 7.5, 150 mM NaCl, 1% NP-40, 0.5% sodium deoxycholate, 2 mM EDTA, 1 mM PMSF, 1 mM Na_3_VO_4_, 4 mM NaF, 20 mM β-glycerophosphate and 5 μl/ml protease inhibitor cocktail (Sigma) and centrifuged at 10,000 g at 4°C for 10 min. The concentration of supernatant was qualified with a BCA protein assay. Finally, the samples were boiled with SDS sample buffer for 5 min, and subjected to SDS-PAGE for Western blot analysis.

### Gel Electrophoresis and western blotting

Samples were separated by SDS-PAGE with 5% stacking gel and 10% separating gel and transferred to a nitrocellulose membrane. After blocking for 1 hour with 5% fat-free milk powder in TBST (10 mM Tris–HCl, 150 mM NaCl, 0.05% Tween-20, pH 7.4), blots were incubated overnight at 4 °C with primary antibodies: 1:1,000 anti-phosphorylated GSK-3α/β (Ser-21/ 9) (Cell Signaling Technology), 1:1,000 anti-GSK-3α (Cell Signaling Technology), 1:1,000 anti-GSK-3β (Cell Signaling Technology), 1:200 anti-β-arrestin2 (Santa Cruz Biotechnology), 1:10,000 anti-α-tubulin (Sigma), 1:1000 anti-GSK-3α/β (Y-279/Y-216) (Millpore), 1:1000 anti-Akt (Abcam), 1:1000 anti-phosphorylated-Akt (Thr-308) (Cell Signaling Technology), and 1:1000 anti-phosphorylated-Akt (Ser-473) (Abcam). After washes, blots were incubated with HRP-conjugated secondary antibodies (Sigma) for 2 hours at room temperature. Immunoactivity was visualized with ECL Western blot detection reagents (GE Healthcare). Data representative of three experimental replicates are shown.

### Statistical analysis

All values are shown as means ± SEM. For comparisons between two groups, t-tests were performed. For comparisons of more than two groups, one-way or two-way ANOVA followed by the Student-Newman-Keuls post hoc analysis was performed.

## Results

### Activation of GABA_B_ receptors increases phosphorylated GSK-3α/β at Ser-21/Ser-9 sites

**Figure 1:**
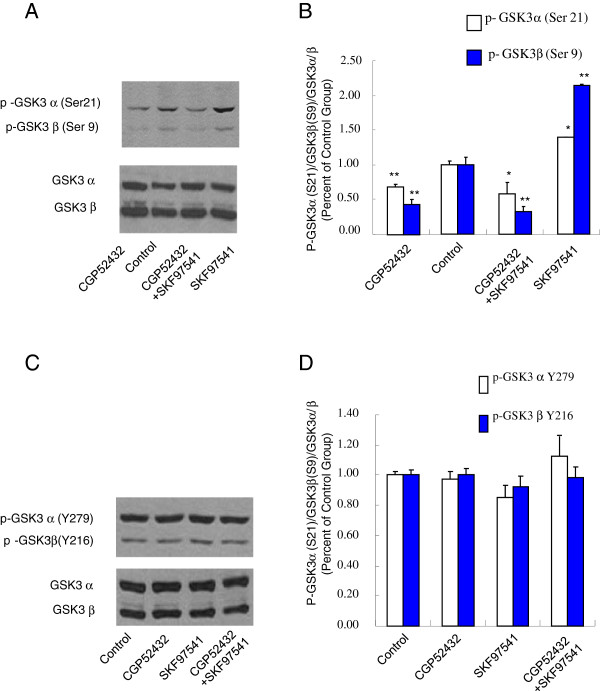
**Activation of GABA_B_ receptors elevates GSK-3α/β phosphorylation at Ser21/Ser 9 sites, but has no effect on phosphorylation of GSK-3α/β at Y279/Y216 sites in HEK293T cells expressing GABA_B_ receptors. A.** Western blot analysis of phosphorylated GSK-3α/β (Ser-21/Ser-9) levels in extract prepared from HEK293T cells transfected with GABA_B_ receptor in the presence of GABA_B_ receptor antagonist and/or agonist. GSK-α/β was used as a loading control. **B.** Densitometric analysis of phosphorylated GSK-3α/β (Ser-21/Ser-9). The intensity of phospho-GSK-3α/β was quantified by densitometry (software: Image J, NIH). **C.** Western blot analysis of phosphorylated GSK-3α/β (Y-279/Y-216) levels in extract prepared from HEK293T cells transfected with GABA_B_ receptor in the presence of GABA_B_ receptor antagonist and/or agonist. GSK-3α/β was used as a loading control. **D.** Densito-metric analysis of phosphorylated GSK-3α/β (Y-279/Y-216). The intensity of phospho-GSK-3α/β was quantified by densitometry (software: Image J, NIH). Data were analyzed by one-way *ANOVA* (*P < 0.05, **P < 0.01, n = 3).

Previous studies have suggested that phosphorylation of GSK-3α/β at Ser-21/Ser-9 sites significantly decreases active site availability, thus inhibiting GSK-3 activity [30]. To investigate whether GABA_B_ receptors are involved in the GSK-3 signaling, we initially tested whether activation of GABA_B_ receptors can modulate the phosphorylation of GSK-3α/β at Ser-21/Ser-9 sites in HEK293T cells expressing GABA_B_R1a and GABA_B_R2 subunits. As shown in Figure 1A, SKF97541 (1 μM, 30 min), a specific GABA_B_ receptor agonist significantly increased GSK-3α/β (Ser-21/Ser-9) phosphorylation, an effect that can be blocked by the GABA_B_ receptor-specific antagonist CGP52432 (10 μM, 30 min). Interestingly, CGP52432 alone induced a small but significant decrease in GSK-3α/β (Ser-21/Ser-9) phosphorylation, suggesting that GABA_B_ receptors may have constitutive activity that is consistent with previous reports [31]. The total expression of GSK 3α/β is not altered among all four groups. The intensity of each protein expression was quantified using dentitometry (Figure 1B). These data suggest that activation of GABA_B_ receptors may enhance GSK-3α/β (Ser-21/Ser-9) phosphorylation, leading to reduced GSK-3 activity.

### Activation of GABA_B_ receptors has no effect on phosphorylated GSK-3α/β at Y-279/Y-216 sites

Previous studies have suggested that phosphorylation at the tyrosine-216 site of GSK-3β or tyrosine-279 of GSK-3α enhances the enzymatic activity of GSK-3. We have shown that activation of GABA_B_ receptors may inhibit GSK-3 activity by enhancing GSK-3α/β (Ser-21/Ser-9) phosphorylation. We then tested whether activation of GABA_B_ receptors can inhibit GSK-3 activity by inhibiting GSK-3α/β phosphorylation at the Y-279/Y-216 sites of GSK-3α/β. As shown in Figure 1C-D, activation of GABA_B_ receptors has no effect on GSK-3α/β (Y-279/Y-216) phosphorylation. These data suggest that GABA_B_ receptors may modulate GSK-3 activity by selectively phosphorylating GSK-3α/β at the Ser-21/Ser-9 sites.

### Activation of GABA_B_ receptors significantly enhances Akt phosphorylation at Thr-308

**Figure 2:**
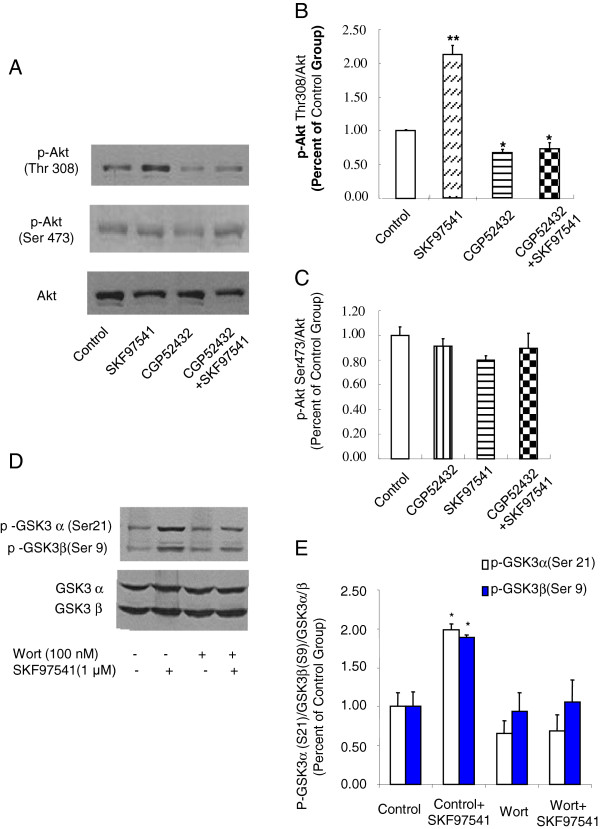
**Activation of GABA_B_ receptors elevates Akt (Thr-308) phosphorylation in HEK293T cells expressing GABA_B_ receptors, which can be blocked with pretreatment of PI3K inhibitor Wortmannin. A.** Western blot analysis of phosphorylated Akt (Thr-308) or Akt (Ser-473) levels in extract prepared from HEK 293T cells transfected with GABA_B_ receptor in the presence of GABA_B_ receptor antagonist and/or agonist. Akt was used as a loading control. **B-C.** Densitometric analysis of phosphorylated Akt (Thr-308) (B) and Akt (Ser-473) (C). The intensity of phospho-Akt was quantified by densitometry (software: Image J, NIH). **D.** Western blot analysis of phosphorylated GSK-3α/β (Ser-21/Ser-9) or GSK-3α/β levels in extract prepared from HEK293T cells transfected with GABA_B_ receptor pretreated with/without Wortmannin (100 nM, 24 h) in the presence or absence of GABA_B_ receptor agonist SKF97541. **E.** Densitometric analysis of phosphorylated GSK-3α/β (Ser-21/Ser-9). The intensity of phosphor- GSK-3α/β (Ser-21/Ser-9) was quantified by densitometry (software: Image J, NIH). Data were analyzed by one-way or two-way ***ANOVA***(*P < 0.05, **P < 0.01, n = 3).

Previous studies have shown that GSK-3α/β activity can be negatively regulated by Akt, a serine/threonine kinase. Dephosphorylation of Akt on its regulatory Thr-308 site leads to a reduction of Akt kinase activity that induces the activation of its substrate GSK-3 [32]. Since we have observed enhanced GSK-3α/β (Ser-21/Ser-9) phosphorylation upon activation of GABA_B_ receptors, we hypothesized that Akt phosphorylation may also be modulated by the activation of GABA_B_ receptors. As shown in Figure 2A-C, GABA_B_ receptor stimulation significantly enhances Akt phosphorylation at Thr-308, but not at Ser-473. These data are consistent with previous studies on dopamine D2 receptor activation of GSK-3 signaling although the direction of effect is opposite.

To further confirm the requirement of Akt activation in the GABA_B_ receptor-mediated GSK-3 signaling, we tested whether phosphatidylinositol 3-kinases (PI3K) inhibitor can block the GABA_B_ receptor-mediated GSK-3 phosphorylation as previous studies have shown blockade of PI3K inhibits Akt activity [33]. As shown in Figure 2D-E, wortmannin (100 nM, 24 h), a PI3K inhibitor, block the effect of GABA_B_ receptor on the phosphorylation of GSK3 at Ser21/Ser9 sites, further confirming the requirement of Akt in the GABA_B_ receptor-mediated GSK-3 signaling.

### GABA_B_ receptors modulate GSK-3α/β phosphorylation through a Gi-protein-independent/ β-arrestin2-dependent pathway

**Figure 3:**
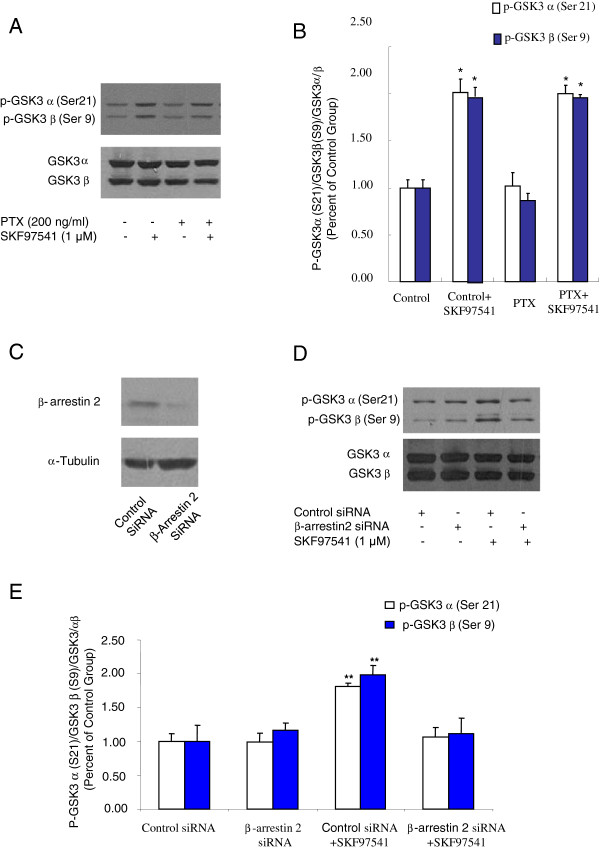
**Effect of GABA_B_ receptors on GSK-3α/β phosphorylation is a Gi protein-independent/β-arrestin2-dependent pathway in HEK293T cells expressing GABA_B_ receptors. A.** Western blot analysis of phosphorylated GSK-3α/β (Ser-21/Ser-9) levels in extract prepared from HEK293T cells transfected with GABA_B_ receptors in the presence or absence of GABA_B_ receptor agonist with/without PTX (200 ng/ml, 14–18 h). GSK-3α/β was used as a loading control. **B.** Densitometric analysis of phosphorylated GSK-3α/β (Ser-21/Ser-9). **C.** Western blot analysis of efficacy of β-arrestin2 siRNA on β-arrestin2 express in HEK293T cells**. D.** Western blot analysis of phosphorylated GSK-3α/β (Ser-21/Ser-9) levels in extract prepared from HEK293T cells transfected with GABA_B_ receptors and β-arrestin2 siRNA or control siRNA in the presence or absence of GABA_B_ receptor agonist. GSK-3α/β was used as a loading control. **E.** Densitometric analysis of phosphorylated GSK-3α/β (Ser-21/Ser-9). The intensity of phospho-GSK-3α/β was quantified by densitometry (software: Image J, NIH). Data were analyzed by two-way ***ANOVA***(*P < 0.05, **P < 0.01, n = 3).

**Figure 4:**
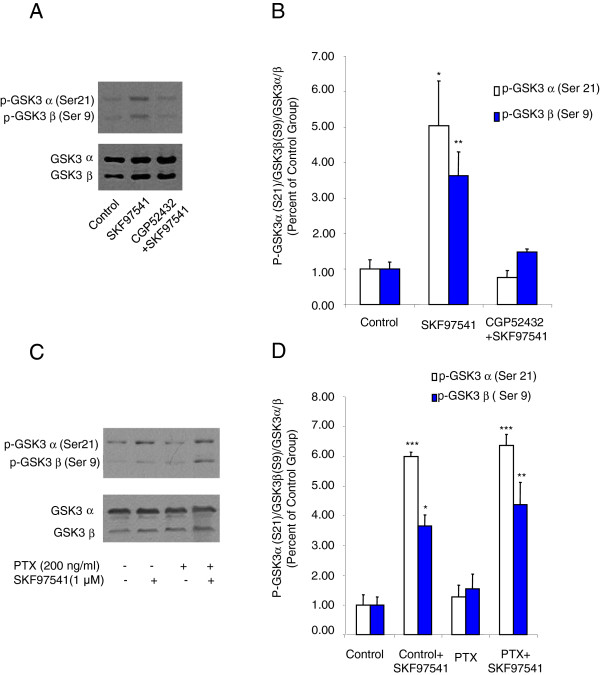
**Activation of GABA_B_ receptors elevates GSK-3α/β phosphorylation in rat hippocampal slices, and this effect can not be blocked by PTX when pretreating primary cultured hippocampal neurons. A.** Western blot analysis of phosphorylated GSK-3α/β (Ser-21/Ser-9) levels in extract prepared from rat hippocampal slices in the presence of GABA_B_ receptor agonist and/or antagonist. GSK-3α/β was used as a loading control. **B.** Densitometric analysis of phosphorylated GSK-3α/β (Ser-21/Ser-9). C. Western blot analysis of phosphorylated GSK-3α/β (Ser-21/Ser-9) levels in extract prepared from rat primary cultured hippocampal neurons pretreated with PTX (200 ng/ml, 14–18 h) in the presence or absence of GABAB receptor agonist SKF97541. GSK-3α/β was used as a loading control. **D.** Densitometric analysis of phosphorylated GSK-3α/β (Ser-21/Ser-9). The intensity of phospho-GSK-3α/β was quantified by densitometry (software: Image J, NIH). Data were analyzed by one-way or two-way ***ANOVA*** (*P < 0.05, **P < 0.01,***P < 0.001, n = 3).

Both GABA_B_ and dopamine D2 receptors are Gi/o-coupled receptors. Traditionally, G-protein coupled receptors exert their effects only via G-protein mediated signaling. However, recent studies have suggested that dopamine D2 receptors can activate the Akt/GSK-3 pathway via G-protein independent/β-arrestin2-dependent signaling [20,27]. Thus, we tested whether Gi/o protein and β-arrestin2 are involved in the GABA_B_ receptor-mediated modulation of GSK-3 signaling. As shown in Figure 3A-B, pre-incubating the cells expressing GABA_B_ receptor or primary cultured hippocampal neurons (Figure 4C-D) with pertussis toxin (PTX) (200 ng/ml, 14–18 h), which uncouples the receptors from Gi/o protein, has no effect on GABA_B_ receptor-mediated modulation of the phosphorylation of GSK-3α/β (Ser-21/Ser-9). This result suggests that GABA_B_ receptors modulate phosphorylation of GSK-3α/β through a Gi-protein-independent pathway.

We then confirmed the efficiency of β-arrestin2 siRNA for knocking-down the expression of β-arrestin2. As shown in Figure 3C, the expression of β-arrestin2 in HEK-293T cells is significantly decreased when transfected with β-arrestin2 siRNA (Santa Cruz Biotechnology), compared to cells transfected with control siRNA. We then measured the phosphorylation of GSK-3α/β (Ser-21/Ser-9) in HEK-293T cells transfected with GABA_B_ receptors and β-arrestin2 siRNA or control siRNA. As shown in Figure 3D-E, activation of GABA_B_ receptors significantly enhanced the phosphorylation of GSK-3α/β (Ser-21/Ser-9) in HEK-293T cells transfected with GABA_B_ receptors and control siRNA, while activation of GABA_B_ receptors failed to alter the phosphorylation of GSK-3α/β (Ser-21/Ser-9) in HEK-293T cells transfected with GABA_B_ receptors and β-arrestin2 siRNA. These data indicate that β-arrestin2 is required for GABA_B_ receptor-mediated modulation of GSK-3 signaling.

### Activation of GABA_B_ receptors increases phosphorylated GSK-3α/β at Ser-21/Ser-9 sites in rat hippocampal slices

To examine the effect of GABA_B_ receptor on GSK-3 signaling in a relevant cellular milieu, rat hippocampal slices were utilized in parallel experiments. As shown in Figure 4A-B, pre-treatment of the hippocampal slices with the GABA_B_ receptor specific agonist SKF97541 significantly enhanced the phosphorylation of GSK-3α/β (Ser-21/Ser-9). Consistent with the data obtained in HEK-293T cells transfected with GABA_B_ receptors, GABA_B_ receptor antagonist CGP52432 abolished the GABA_B_ receptor effect on phosphorylation of GSK-3α/β (Ser-21/Ser-9). These data further confirm that GABA_B_ receptors are involved in GSK-3 signaling.

## Discussion

**Figure 5:**
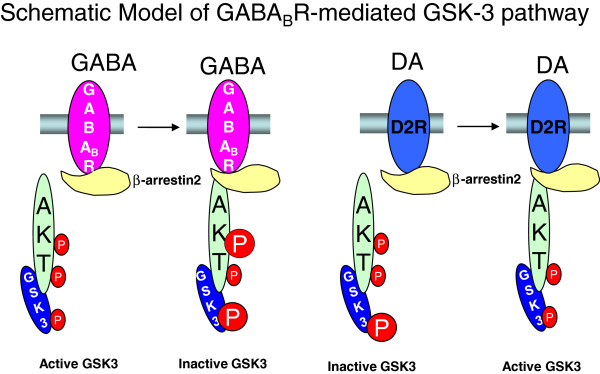
**Schematic model of GABA_B_ receptor mediated GSK-3 signaling.** Activation of GABA_B_ receptors enhances the phosphorylation of Akt and GSK-3α/β, which inactivates GSK-3 signaling. Activation of D2 receptors has the opposite effect as previously reported [37].

Our findings suggest that activation of GABA_B_ inhibits GSK-3 signaling through a β-arrestin2-dependent pathway (Figure 5). This pathway involves the upregulation of Akt phosphorylation at Thr-308 and GSK-3α/β phosphorylation at Ser-21/Ser-9. As a G-protein coupled receptor (GPCR), the GABA_B_ receptor was thought to exert its effects via coupling to pertussis toxin (PTX) sensitive Gi/o proteins, that in turn regulate voltage-gated Ca^2+^ (Ca_V_) or G protein-gated inwardly rectifying K^+^ (GIRK) channels, and inhibit adenylyl cyclase. However, our results suggest that activation of GABA_B_ receptor modulates GSK-3 signaling in a G-protein independent manner, as PTX failed to block the GABA_B_ receptor effect on GSK-3α/β phosphorylation. Interestingly, previous studies have shown that activation of dopamine D2 receptors, which are also Gi/o coupled GPCRs, similarly modulate GSK-3 signaling in a β-arrestin dependent pathway. However, the D2 receptor effect on GSK-3 is opposite to the GABA_B_ receptor effect. Activation of D2 receptors leads to β-arrestin2 recruitment to the D2 receptors and formation of a β-arrestin2-scaffolded protein complex that includes protein phosphatase 2A (PP2A), Akt and GSK-3α/β. PP2A dephosphorylates Akt at Thr-308 which subsequent activation of GSK-3α/β as a consequence of dephosphorylation of GSK-3 at Ser-9 and 21 [34]. It is worth noting that both receptors modulate GSK-3 signaling by changing the Akt phosphorylation at Thr-308 site and GSK 3α/β phosphorylation at Ser-21/Ser-9 sites. The fact that both GABA_B_ receptor agonists and D2 receptor antagonists exert antipsychotic effects [5,35], together with previous findings that antipsychotics are potent antagonists of the dopamine-induced recruitment of β-arrestin2 to the D2 receptors [36], suggests that inhibition of GSK-3 activity may be a molecular mechanism through which GABA_B_ receptor agonists have antipsychotic effects.

Previous studies have suggested that GPCRs can signal without an external chemical trigger, i.e., in a constitutive or spontaneous manner [36]. For example, dopamine D5 receptors enhance cAMP accumulation without agonist stimulation [38,39]. Consistent with this idea, GABA_B_ receptors also display constitutive activity as we observed a significant decrease of GSK-3α/β phosphorylation at Ser-21/Ser-9 sites treated only with the GABA_B_ receptor antagonist CGP52432. The general physiological purpose of such basal activity may be to permit bi-direction control of receptor activity. With constitutively active pathways, the output can be either increased or decreased from a mid-range level.

GSK-3 is a multi-functional serine/threonine kinase. Its activity is regulated negatively by the phosphorylation of Ser-9 and positively by the phosphorylation of Tyr-216, a GSK-3β auto-phosphorylation site required for regulating its activity. Previous studies have shown that GSK-3β phosphorylsation at Tyr-216 can be prevented by its interaction with DISC1 (Disrupted-in-schizophrenia-1 protein) [40]. Thus, it is possible that GABA_B_ receptors inhibit GSK-3 activity through direct inhibition of GSK-3β phosphorylsation at Tyr-216 site. However, our results indicate that activation of GABA_B_ receptors has no effect on GSK-3β phosphorylation at Tyr-216. Interestingly, this data is also consistent with the dopamine D2 receptor effect on GSK-3 phsphorylation as activation of D2 receptor also has no effect on GSK-3β phosphorylation at Tyr-216.

Available evidence suggests that antipsychotic drugs exert their antipsychotic effects in schizophrenia through the blockade of dopamine D2 receptors (D2R) or D2R in combination with the serotonin receptor 2A (5-HT_2A_R) [25,26,41]. GABA_B_ receptors and D2R belong to the super family of G-protein coupled receptors (GPCRs) that exert their biological effects via intracellular G protein-coupled signaling cascades [42-45]. D2Rs display a complex pattern of signal transduction via their coupling to the Gi/Go protein. Previously, D2Rs were known to stimulate a number of signal transduction pathways including the inhibition of adenylate cyclase activity, PI (phosphatidylinositol) turnover, potentiation of arachidonic acid release, inwardly rectifying K^+^ and Ca^2+^ channels and mitogen activated protein kinases [43]. Recently several studies have suggested that D2R can activate the Akt/GSK-3 pathway via β-arrestin2-dependent signaling. D2R-mediated Akt/GSK-3 regulation involves the recruitment of β-arrestin2 to the D2R and the formation of signaling complexes containing β-arrestin2, protein phosphatase 2A (PP2A) and Akt. Formation of this protein complex leads to specific dephosphorylation/inactivation of the serine/threonine kinase Akt on its regulatory Thr-308 residue but not the second regulatory Ser-473 residue [23,27,43] the inactivation of Akt, in response to DA stimulation, leads to a reduction of kinase activity and a concomitant activation of its substrates GSK-3α (Ser-21)/β (Ser-9) since both are negatively regulated by Akt [20]. Interestingly, D2R-mediated modulation of GSK-3 signaling targets the same phosphorylation sites as GABA_B_ receptors, but the functional effects are the opposite. The fact that antipsychotics block D2R and also antagonize the agonist-induced recruitment of β-arrestin2 to D2R [29], supports our contention that GABA_B_ receptor-mediated inhibition of GSK-3 signaling may be a target for the development of novel antipsychotic medications.

## Competing interests

The authors declare that they have no competing interests.

## Authors’ contributions

FFL carried out all the Western Blot analysis. PS carried out all the cell transfection, data analysis and prepared all the figures. FL and ZJD supervised the project and wrote the manuscript. All authors read and approve the manuscript.
